# Epistaxis and Hemorrhage from a Wound in the Superior Coronary Successfully Treated by Compression upon the Artery

**Published:** 1888-11

**Authors:** B. Frank Humphreys

**Affiliations:** Hawkins, Tex.


### EPISTAXIS AND HEMORRHAGE FROM A WOUND IN THE SUPERIOR CORONARY SUCCESSFULLY TREATED BY COMPRESSION UPON THE ARTERY.

By B. FRANK HUMPHREYS, M. D., HAWKINS, TEX.

On June 26, 1888, Henry Partain, at 30, a sawyer at a mill, one mile from this place, while repairing a circular saw, fell upon the same in such a manner that one tooth of the saw struck him upon the right side of the nose, but not with sufficient force to crush the nose to any great extent. Another tooth of the saw struck him in the region of the levator labii superioris, on the right side, near its origin or lower aspect, ploughing its way downwards through the fascia, thereby rupturing the superior coronary artery, which, on that account, discharged most of its current of blood inwardly, through the rent made in the upper lip, the current being thrown against the alveo ar process of the superior maxillary, flowed in a constant stream from the mouth, while another stream issued from the right side of the nasal orifice.

The man did not suppose at first that he was so seriously wounded, and remained at the mill an hour after the accident, trying to check the blood. Finding, however, that he could not stay the constant flow, he then went to his residence, only two hundred yards distant. His wife and mother insisted that a physician be called at once; but, strange to say, the man still persisted in thinking that by the application of cold water they could check the incessant hemorrhage. Two precious hours were thus wasted in futile attempts to accomplish that end. When it became apparent that their efforts were powerless—then I was called to the case. It was an hour later before I saw the wounded man, who had been bleeding profusely four hours, perhaps.

He was lying upon a bed, near the edge, in a semi-recumbent position, supported partly on his left elbow, with his head inclined so that the blood could flow into a basin placed upon the floor. In this position the blood had been flowing for three hours, perhaps, not only into the pan, but while it was being removed and emptied, it flowed upon the floor in pools. It was, indeed, a bloody scene. The man was exceedingly pale and nervous, face swollen, eyes closed, pulse at the wrist almost imperceptible. He was so weak that he either could not or would not speak a word, and all the indications seemed to point to a speedy dissolution due to exhaustion.

I had no one to assist me except the patient's wife and feeble mother, both of whom were exhausted through alarm; and, so excited, they were poorly able to render me the necessary assistance in this emergency. Futhermore, it was quite apparent that time was too precious to be lost in attempting to accomplish what seemed an unpromising undertaking at that time, (securing and ligating the artery,) owing to the contused and lacerated nature of the wound, the exhausted condition of the patient, lack of assistance, etc. Therefore placing the palmar surface of my thumbs near the wound, immediately over the artery, a sufficient degree of pressure was made to check the flow of blood, not only from the mouth, but from the nose likewise. This at once brought hope out of despair.

Then the cork of an ounce quinine jar was enveloped in a bit of soft linen and placed over the artery where the thumbs had been placed, and retained in position by an assistant until I brought the edges of the wound together by stitches and covered the same with antiseptic dressings. Then a bandage was placed over the cork compresss extending underneath the right ear, thence backwards over the occipital region in a horizontal direction to the mastoid process of the left side, thence forward beneath the left ear and horizontally across the upper lip, passing again over the wound and cork compress as before, until the bandage was doubled upon itself several times with sufficient tension to prevent further hemorrhage. Another bandage, attached to the first in front of the right ear, passed upwards over the head, thence downwards in front of the left ear, where it was attached to the horizontal bandage, and reversed two or three times, thereby securing the first bandage in position.

The patient was now placed in a comfortable position in bed and allowed to rest until he might regain his strength in a measure, when I intended, if necessary, to ligate the artery. He was doing so well at this time, his wife and mother desired that he be permitted to remain undisturbed while he was so exhausted, and if it should be deemed nesessary for further treatment on the morrow, I should be notified at once. The patient's health was greatly impaired at the time of the accident, on account of having just recovered from a severe attack of pneumonia, which made the accident a serious one, in consequence of the hemorrhage that resulted through delay. Nothing further was heard from the patient for several days, when I heard that he was doing well, and within a week he had resumed his position at the saw mill, the treatment having accomplished all that could be desired.

The details of the foregoing case have been given, not only to illustrate a singular and successful method of treating a somewhat unusual accident, but rather to emphasize the value and importance of compression over the superior coronary arteries in obstinate cases of ordinary epistaxis, when the usual means fail—and fail they do, not infrequently. Furthermore, it is believed by the writer that the method adopted, in the case reported, was the speediest and most available means of relief to the patient, whose life was imperiled by even a short delay in checking further hemorrhage.

Dr. B. N. Herring read a paper to the Wilson County (N. C.) Medical Society, December, 1887, on "Little Things in Medicine" which was published in the *North Carolina Medical Journal*, and reproduced in the SOUTHERN MEDICAL RECORD for June, 1888. The author gives some very good suggestions in regard to a few things which, if not serious, are sometime more or less perplexing to the practitioner. The editor of the SOUTHERN MEDICAL RECORD, commenting on the paper referred to, suggested "a more excellent way" to accomplish some of these "little things in medicine"—and the editor might justly have commented upon another item of the author's paper.

Dr. Herring devotes nearly a page to the consideration of epistaxis. After alluding to the plugging of the posterior nares, and criticising the ordinary methods, in language unusually severe, he says: "As laid down in the books on surgery, it is barbarous, painful, unscientific, and only accomplishes its object by indirect means, . . . and the doctor who would apply Monsel's Solution to the nasal fossa, ought to be indicted for malpractice." Then follows a volley of severe denunciations against "Bellocq's canula, plugs of sponge or cotton, with possible pyemia and death (!) all to stop a little blood from the nose;"—referring to such methods and practitioners of the same, as "blind imitators" "without thought," "more like apes or ignorant negroes," he tells the aforesaid class "how to do it," as follows: "Take a piece of tough, raw, fat salt-pork, cut it wedge-shaped, four inches long, half an inch thick and three-quarter of an inch wide. Force it into the bleeding cavity—*clear through into the pharynx*—and the work is done. It is antiseptic, painless, and never fails to stop the hemorrhage. It is easily removed, and the lookers on are often solicitious to know what you put on the meat to produce such marvelous results. A hint to the wise (quack) is sufficient." Comment to the foregoing brief quotations is certainly unnecessary. Physicians will probably continue to practice the methods of our standard authors on surgery, however "barbarous, painful and unscientific" they may seem to those who cannot offer something better and more reliable.

Dr. J. Robinson, (*Therapeutic Gazette*, August, 1887), in a short communication on the treatment of epistaxis, says: "Now, it is a well known fact to anatomists and others that the hemorrhage in the vast majority of cases proceeds from the septum nares, and is supplied by a branch of the superior coronary, a branch of the facial, which ramifies upon the septum nares. It enters the opening of the nose just below the alo-nasi, crossing the superior maxillary bone at that point. Now, in a practice of nearly thirty years, I have had many cases of epistaxis, and have never in a single case failed to arrest the bleeding by compression of the aforesaid artery, with the finger applied over its tract, making firm pressure against the bone. This will arrest the bleeding nine hundred and ninety-nine cases in a thousand. I have been called to see cases where other physicians had plugged the nostrils and injected solutions of ferri per sulphas, ice water, etc., without benefit, and have at once arrested all hemorrhage instantly by the above simple means."

The foregoing very practical suggestions of Dr. Robinson are timely and worthy of note. I would only add that when there is reason for apprehending the reccurrence of the bleeding, it would probably be better to apply the cork compress and bandage as described herein, with instructions to lessen or increase the tension of the bandage according to indications.

In this connection it may be noted that M. Verneuil, of Paris, has read a communication at the Academy of Medicine on the treatment of certain grave forms of epistaxis by counter-irritation over the region of the liver, when the epistaxis is symptomatic of cirrhosis or other disease of that organ, nephritis, or heart-disease. M. Verneuil's treatment, (three cases), which was immediately and permanently efficacious, consisted of the application over the region of the liver of a large blister.—*London Lancet*, April 30, 1888.

**Rhus Poisoning** is said to yield quickly to the local application of fluid extract of grindelia robusta.

